# Palatal Protective Stents Prevent Oro-Nasal Fistulas after Surgery for Velopharyngeal Insufficiency: A Preliminary Report

**DOI:** 10.3390/dj6030029

**Published:** 2018-07-04

**Authors:** Kongkrit Chaiyasate, Pablo Antonio Ysunza, John Spolyar, Rafaella Genova, Peter Andrade

**Affiliations:** 1 Ian Jackson Craniofacial and Cleft Palate Clinic of Beaumont Hospital, Royal Oak, MI 48073, USA; kongkrit.chaiyasate@beaumont.edu (K.C.); yours2make@yahoo.com (J.S.); drplastics@gmail.com (P.A.); 2 Oakland University William Beaumont School of Medicine, Rochester, MI 48309, USA; rgenova@gmail.com

**Keywords:** cleft palate, surgery, fistula, speech, resonance

## Abstract

Background: One of the potential complications of surgery for velopharyngeal insufficiency (VPI) is postoperative oral-nasal fistula (ONF). Reported rates vary from 0 to 60%. Several factors are on account of these disproportionate rates. Objective: The purpose of this study was to describe the use of a palatal protective stent (PPS) to preserve the VPI repair surgical site and to study its effectiveness for decreasing the incidence of postoperative ONF. Materials and Methods: A retrospective study was carried out. All patients undergoing surgery for VPI with complete preoperative and postoperative evaluations including at least one year follow up after surgery from 2012 to 2016 were studied. Some of the patients were operated on using a pre-molded palatal protective stent (PPS). Twenty-seven patients were included in the study group. Most of the patients underwent a customized pharyngeal flap according to findings of imaging procedures. The remaining cases underwent a Furlow palatoplasty. Twelve patients were operated on using PPS. Results: There were no surgical complications during the procedures. ONF was detected in four of the patients operated on without PPS. None of the patients undergoing surgery using PPS demonstrated ONF. All fistulas were located at the soft/hard palate junction. VPI was corrected in 92% of the cases. Conclusion: Although only a reduced number of cases were studied, these preliminary results suggest that using PPS during surgical procedures for correcting VPI is a safe and reliable tool for preventing ONF.

## 1. Introduction

Cleft palate surgery has been performed for nearly two centuries. The procedure was originally described by Dieffenbach in 1837. He utilized palatal relaxing incisions for enhancing closure of the anatomical defect [1]. Palatoplasty techniques have been continuously improving and are continually evolving. Some of the reasons for the multitude of palatoplasty techniques have been to prevent palatal fistula formation, reduce restrictive facial growth patterns, and restore velopharyngeal sphincter function during speech. Furthermore, a successful palatoplasty should accomplish the compartmentalization of the oral and nasal cavities. The procedure should also provide sufficient velum length, and the re-orientation of the fibers of the *levator veli palatini* muscle in a transverse fashion for enhancing its sling function [2].

Velopharyngeal insufficiency (VPI) is the inability of the velopharyngeal sphincter, including the velum, the lateral pharyngeal walls and the posterior pharyngeal wall for achieving a complete seal between the nasal cavities and the rest of vocal tract. The normal function of the velopharyngeal sphincter achieves a resonance balance during speech. An adequate velopharyngeal seal increases oral air pressure during swallowing and during the articulation of high intraoral pressure consonant phonemes. VPI results in nasal regurgitation of liquids, recurrent serous otitis media and hypernasal speech. Barr et al. reported that children with VPI and their families can have a significantly negative quality of life [3]. VPI persists after initial palatal repair in approximately 20–30% of the cases [4]. Although speech pathology treatment is effective for correcting compensatory articulatory behaviors associated with VPI, the defective velopharyngeal function has to be corrected surgically or by using a pharyngeal prosthesis. Currently, there is still controversy concerning which is the best surgical technique for correcting VPI. Because velopharyngeal sphincter anatomy and physiology is highly variable from individual to individual, VPI surgery must be contrived to each individual case depending on the specific characteristics of each case. The only approach for assessing velopharyngeal function during speech is the use of imaging procedures such as videonasopharyngoscopy (VNP) and multiplanar videofluoroscopy (MPVF). The most common surgical techniques for correcting VPI are pharyngeal flap, sphincter pharyngoplasty, secondary palatoplasty and velopharyngeal augmentation procedures [5].

Palatal fistulae are defined as a breakdown of the palatoplasty site resulting in an epithelialized communication between the oral and nasal cavities [6]. Oral-nasal fistulae (ONF) are the most common complications following palatoplasty [7]. Widely reported in the literature, fistula rates vary from 0% to 60% [8]. The preponderance of different surgical techniques, cleft width, timing of repair, and the technical ability of the surgeon can explain the vast disparity of reported incidences of fistula formation. ONF can be asymptomatic but it can also lead to impaired speech patterns and nasal regurgitation. Several procedures have been reported for closure or obturation of ONF including cauterization, prosthetic obturation or surgical techniques. In 1978, Berkman proposed early non-surgical repair of palatal fistulae using temporary vinyl retainer-type devices to cover and close the defect [8]. Gelfoam interposition and the use of fibrin tissue sealant during palatal repair have also been proposed as being useful for preventing postoperative fistulae [9,10].

The purpose of this study was to describe the use of a palatal protective stent (PPS) for preserving the VPI repair surgical site and to study its effectiveness for decreasing the incidence of postoperative ONF.

## 2. Materials and Methods

### 2.1. Patients

After obtaining Institutional Review Board approval, the charts of patients undergoing VPI repair surgery with and without the application of the PPS by a single surgeon (first author of this paper) were reviewed. The study period was from January 2012 to December 2016. The study group included 27 patients who presented with VPI either as a consequence of an occult subtotal cleft of the secondary palate, A. K. A. “submucous cleft palate” or persistent postoperative VPI after a repair of a total cleft palate. All these patients underwent further surgery for correcting residual VPI. The specific surgical procedure in each case was selected and performed according to findings of VNP and MPVF as previously reported [11,12]. It should be pointed out that patients with compensatory articulation (five patients) received speech pathology treatment before the imaging procedures until they were able to repeat the speech sample with adequate articulation placement. These patients continued receiving speech pathology treatment after the surgical procedure. Twelve patients underwent VPI surgery with the application of a PPS whereas 15 patients underwent surgical repair of VPI without using a PPS. The decision of using PPS or not was not randomized. There were different reasons for not using PPS including parent decision, insurance coverage, difficulties for going to the Orthodontics department for preoperative molding or technical difficulties or lack of compliance during molding, etc.

Out of the total study group of 27 patients, eighteen patients presented with unilateral cleft lip and palate (UCLP), two patients presented with bilateral cleft lip and palate (BCLP), two patients presented with complete cleft of the secondary palate (CP) and five patients presented with 22q11.2 deletion, demonstrated by a positive FISH test. These 5 patients had occult subtotal clefts of the secondary palate, A. K. A. “submucous cleft palate” [11].

All patients with syndromic subtotal palatal clefts were operated on by a pharyngeal flap which was customized according to imaging procedures (VNP and MPVF) as described previously [11,12]. PPS was used in all these cases.

Eighteen patients with UCLP and two patients with BCLP which had been repaired when they were infants and with persistent VPI were operated on by a pharyngeal flap which was customized according to imaging procedures.

Out of 20 patients with non-syndromic UCLP or BCLP, four patients underwent adenoidectomy and tonsillectomy (T and A) in preparation for the pharyngeal flap. The pharyngeal flap surgery was performed 3–4 months after T and A.

All 5 patients with syndromic sub-total clefts of the secondary palate underwent T and A in preparation for the pharyngeal flap.

Two patients with CP were operated on using Furlow’s “Z” palatoplasty [13].

The age of the patients ranged from 3 to 11 years old at the time of the surgical procedure for correcting VPI. Median age was 5.5.

There were no complications during the surgical procedures or during the immediate postoperative period.

All patients were followed for at least one year. Correction of VPI was considered when mean nasalance during conversational speech (reading or repeating words from the Rainbow Passage) was <35% and a postoperative MPVF demonstrated complete closure of the velopharyngeal port either by velopharyngeal sphincter movements in the cases operated on by secondary palatoplasty or by occlusion of both ports of the flap by movements of the lateral pharyngeal walls during speech. 

ONF was defined as a defect in the palate after VPI repair not attributed to dehiscence of the surgical site. Intraoral examinations of the patients were performed at 1 week, 3 weeks, 6 weeks, 3 months, 6 months, and one year postoperatively.

### 2.2. Surgical Procedures

#### 2.2.1. Pharyngeal Flap

A superiorly based pharyngeal flap and intravelar veloplasty were performed in 23 of the cases including five cases with syndromic sub-total clefts of the secondary palate. As mentioned herein the flap was customized according to preoperative findings of imaging procedures as previously described [11,12].

The flap was performed according to the following technique: After placement of a Dingman retractor, the soft palate was split in the midline. The *levator veli palatini* muscle was sharply dissected off of the posterior shelf of the palate. The muscle was then sharply separated from the nasal and oral mucosa. The posterior pharynx was then incised and deepened to the level of the prevertebral fascia to create a superior based pharyngeal flap. The caudal end of the pharyngeal flap was then sutured to the nasal mucosa using 3-0 Monocryl. The muscle fibers were reoriented in a transverse fashion in the posterior third of the palate and secured to the pharyngeal flap with a 4-0 Monocryl. The oral lining was re-approximated with 4-0 Monocryl. The pharyngeal defect was closed with 4-0 Vicryl.

#### 2.2.2. Secondary Furlow’s “Z” Palatoplasty

Two patients with CP exhibited a small posterior velopharyngeal closure gap of <4 mm as demonstrated by VNP and MPVF. Thus, in these cases a secondary Furlow’s “Z” palatoplasty [13] was performed. A PPS was used in one of the cases. For the palatoplasty, the soft palate was divided in the midline. A standard double opposing “Z” palatoplasty flaps were designed. The posteriorly based flaps captured the muscular complex while the anterior flaps contained mucosa only. The flaps were then transposed in order to facilitate the sling function of the *levator veli palatini* muscle which was being reoriented transversely. The flaps were sutured together with a 3-0 Vicryl suture.

#### 2.2.3. Protective Palatal Stent

**Figure 1:**
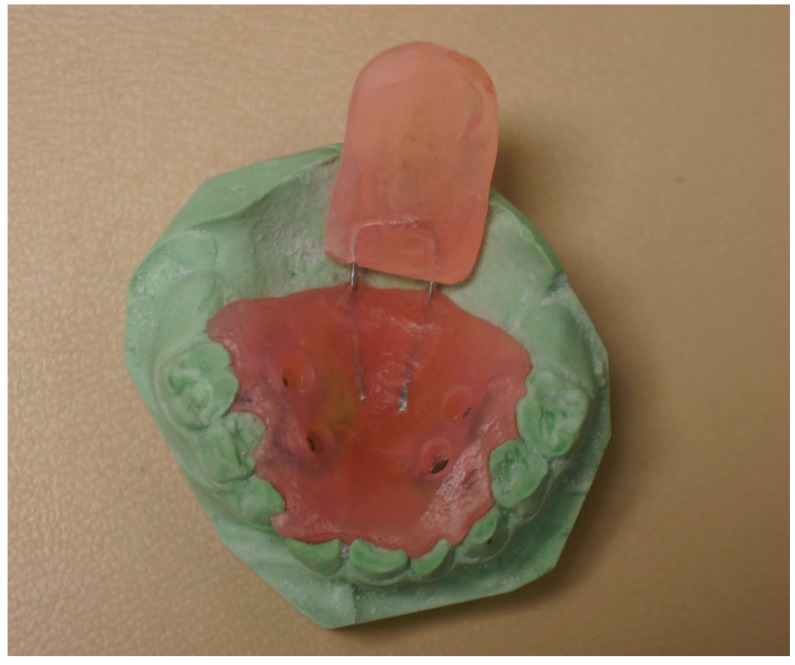
PPS fabricated from a dental impression.

**Figure 2:**
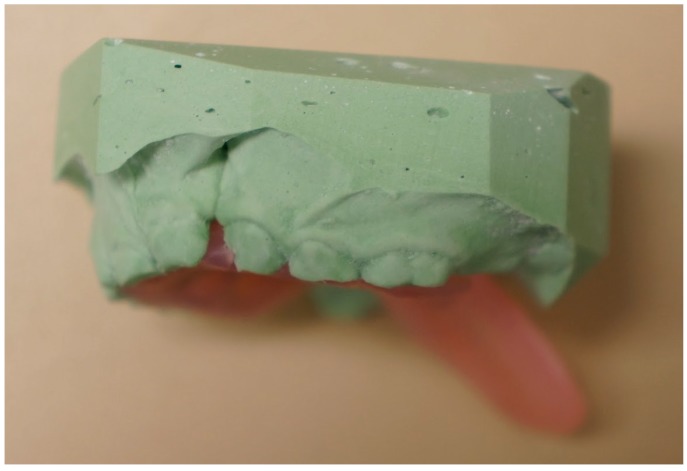
Lateral view of dental impression with the posterior extension of the PPS to cover pharyngeal flap.

**Figure 3:**
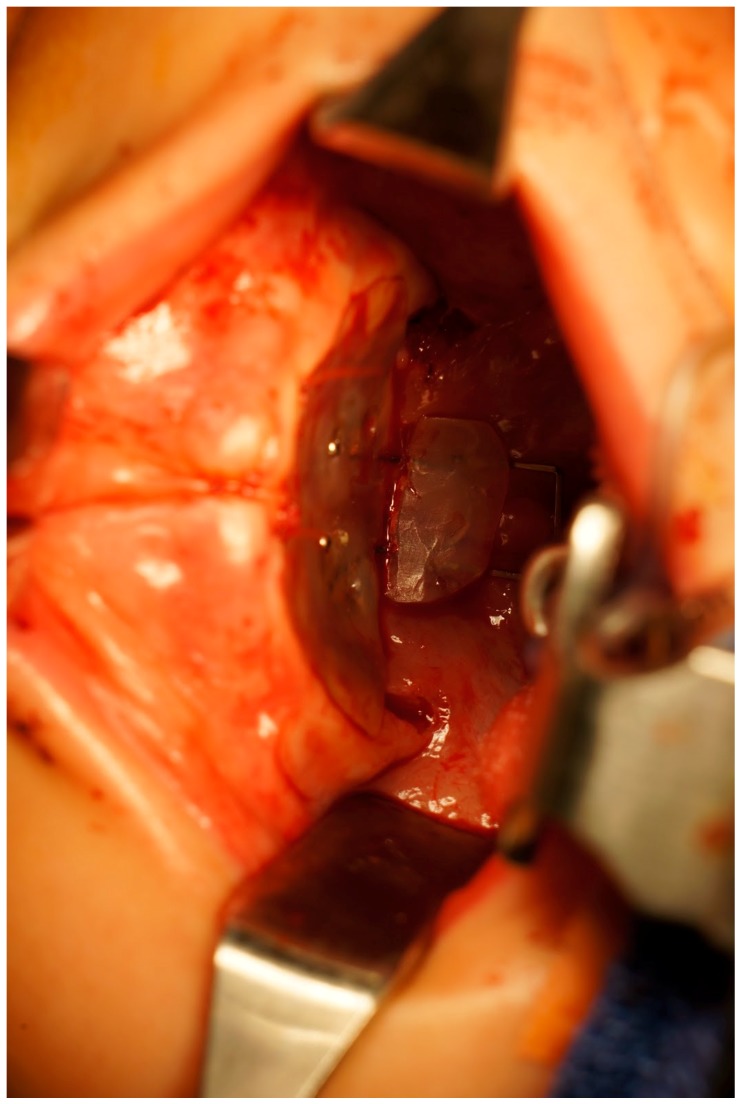
PPS fixated to the hard palate with the posterior extension covering the pharyngeal flap.

The PPS has to be molded preoperatively by the Orthodontics department of our institution. Acrylic resins (Figure 1) were used. After the surgical procedure (either a pharyngeal flap or a secondary palatoplasty) had been completed, the PPS was placed over the palate to ensure a proper fit. The optimal PPS would cover the hard and soft palate along with an extension to protect the pharyngeal flap whenever necessary (Figure 2). If the posterior extension of the PPS was too long it would be sharply fashioned in the operating room and re-applied. Once adequate dimensions were obtained, the PPS was secured rigidly to the hard palate with a 4 screw fixation (Figure 3).

Postoperatively, the patient would be placed on a soft diet, age appropriate, until the device was removed. The PPS was explanted at 2–3 weeks postoperatively in the operating room. At this time, a comprehensive oral exam was performed in order to assess for any fistula formation. 

## 3. Results

This retrospective study included 27 patients who underwent surgical procedures for correcting VPI with and without the use of PPS. Patients were assessed for occurrence of ONF for at least 1 year follow up. The PPS was removed at 3 weeks postoperatively in 11 (91.7%) patients. The PPS became dislodged in 1 (8.3%) patient and had to be removed 2 weeks after the surgical procedure. There were no cases (0%) of ONF formation in patients undergoing VPI repair using the PPS as compared to 4 (27%) patients with ONF in the control group including patients in which PPS was not used. All 5 patients with ONF had UCLP. These patients underwent a customized pharyngeal flap surgery. All fistulas were located at the soft/hard palate junction. There were no (0%) infections, excessive bleeding, hematomas, wound dehiscence, or airway compromise in any of the patients.

A complete speech assessment including nasometry and postoperative MPVF demonstrated correction of VPI in 25 of the cases. Two cases demonstrated postoperative Mean Nasalance >35% (40% and 41%). These two cases had a UCLP and underwent pharyngeal flap surgery. PPS was not used in these two cases. Postoperative MPVF demonstrated that the flap was situated lower than originally planned and this was attributed to inferior migration of the flap in the postoperative period. Thus, VPI was corrected with a 92% success rate.

## 4. Discussion

The presence of VPI demands surgical treatment. There have been a multitude of different techniques described in the literature to repair VPI. Pharyngeal flap, sphincter pharyngoplasty, and Furlow palatoplasty are the most commonly used surgical procedures and they have been long been employed for treating VPI. It is the burden of the provider to determine which surgical procedure is the most adequate in each case. In this paper, most patients were treated with customized pharyngeal flaps. 

The results of this study suggest that the use of PPS during surgical correction of residual VPI after palatal repair is a safe and effective procedure for preventing PPF in cases of surgery for VPI.

Pharyngeal flaps were originally described in 1876 by Shoenborn [14]. The flap provides a static central obstruction with open lateral ports.

The majority of the patients studied for this paper underwent pharyngeal flap surgery which is one of the most common surgical procedures for correcting residual VPI after palatoplasty. While palatoplasty closes the cleft palate, it can leave a contracted and less mobile soft palate which accounts for a deficient velar excursion.

Only superiorly based pharyngeal flaps were performed. A superiorly based flap can be easily elevated with adequate exposure. Inferiorly based pharyngeal flaps have their own shortcomings such as limited length which leads to tethering [15]. The flaps were customized individually according to findings of VNP and MPV.

In this group of patients, when pharyngeal flaps were performed, an additional intravelar veloplasty was performed as described by Sommerland [16]. In our experience, it seems beneficial to reconstruct the soft palate even if the patient had a previous palatoplasty. As a surgical finding in cases of pharyngeal flap procedures, after a palatoplasty several patients show the *levator veli palatini* muscle complex anteriorly migrated to an ineffective position due to cicatricial contracture. A procedure to re-position the muscle complex to the posterior third of the soft palate can be useful in cases of pharyngeal flap surgery.

Furlow originally described his double opposing Z-plasty technique in 1986 [13,17]. Since he published his work, his technique has also been advocated for the treatment of VPI. We arbitrarily chose to utilize a Furlow palatoplasty in patients with a sagittal deficiency of 4 mm or less. One of our concerns was compromising the blood supply to the soft palate if a different palatoplasty had been done prior. Hsu and colleagues showed in their study that the soft palate was not adversely affected with the use of a re-do double opposing Z palatoplasty [18]. They proposed that delaying the double opposing Z palatoplasty one year would mitigate any blood supply complications. Furthermore, they argued that the act of lengthening the velum more so than re-orienting the musculature was the catalyst for improved VPI in all 13 patients in their series. Although we do not disagree with this notion, we do acknowledge that both can be accomplished if the Furlow procedure is done correctly and meticulously.

The angst with ONF formation has led to a multitude of different techniques in VPI surgery. Winters and colleagues described the use of a standardized protocol in which they incorporated acellular dermal matrix into some of their patients [19]. They reported ONF rate of 9% which was reduced to 4% due spontaneous healing of some of the fistulas. Although a direct correlation could not be established, one can surmise that the additive layer of an ADM may help deter ONF development. Regardless, this study shows that sound, consistent surgical techniques can yield low ONF rates. Basha and colleagues used Von Langenbeck repair and fibrin glue adhesive between the oral and nasal layers as a method to decrease ONF [20]. They reported a 0% ONF rate in their fibrin glue treatment group vs 30% ONF rate in the control group. They proposed that the fibrin glue acts to close the dead space between the nasal and the oral mucosa which leads to less incidence of ONF formation.

Although the etiology of ONF is multifactorial, we contend that one of the driving forces for ONF formation is the repetitive trauma to the surgical site post repair. Constant irritation leads to increased inflammation which is conducive for the emergence of an ONF. For this precise reason, a PPS was developed by the Surgery and Orthodontics departments of the Beaumont Craniofacial and Cleft Palate Clinic in order to safeguard the incision from traumatic insults such as the tongue, food bolus, foreign bodies, fingers etc. The PPS is prefabricated pre-operatively and adjusted accordingly intra-operatively. The fashioning and fixating of the PPS added only approximately 8 min to each case. Admittedly, we anecdotally concede that the first 3 weeks are the most critical to protect the surgical site. In one patient, the PPS had to be removed at 2 weeks because of loss of stability. However, this did not precipitate the formation of an ONF.

The technology for the creation of the PPS dates back to the mid nineteenth century. Charles Stent, a dentist, created dental impressions by using gutta-percha, stearine, and talc [21]. Today, the word ‘stent’ is ubiquitous in the medical arena. In fact, cardiac and biliary stents bear his name due to his contributions. Hence, the longevity and safety profile of acrylic stents and has been well validated over the past two centuries.

Certainly, given the retrospective nature of our series, our study has several limitations. However, the results seem promising. It will be necessary to carry out prospective studies in which the patients can be randomly assigned to a control group and a PPS group. Moreover, as reported previously, it will be best to stratify patients into groups with less variability and more homogeneity such as assigning the patients to different groups including various age ranges, patients with UCLP, patients with BCLP, syndromic patients, and so on and so forth. Lastly, increasing the number of patients in the study would be useful for further enhancing the power of these preliminary results [22].

## 5. Conclusions

To our knowledge, this is the first report about prefabricating and applying a PPS in cases of surgical procedures aimed to correct VPI. The preliminary results of this study, although limited, seem promising. None of the patients treated with a PPS developed ONF. The technique is quick, cost effective, and easily reproduced. However, further studies are needed to confirm these preliminary results.
